# TRPC3 Is Downregulated in Primary Hyperparathyroidism

**DOI:** 10.3390/ijms25084392

**Published:** 2024-04-16

**Authors:** Emilie Kirstein, Dirk Schaudien, Mathias Wagner, Coline M. Diebolt, Alessandro Bozzato, Thomas Tschernig, Colya N. Englisch

**Affiliations:** 1 Institute for Anatomy and Cell Biology, Saarland University, 66421 Homburg, Germanycolya.englisch@uni-saarland.de (C.N.E.); 2 Fraunhofer Institute for Toxicology and Experimental Medicine, 30625 Hanover, Germany; 3 Department of Pathology, Saarland University, 66421 Homburg, Germany; 4 Department of Otorhinolaryngology, Head and Neck Surgery, Saarland University, 66421 Homburg, Germany; alessandro.bozzato@uks.eu

**Keywords:** TRPC3, TRPC6, CaSR, parathyroid gland, primary hyperparathyroidism, human, immunohistochemistry

## Abstract

Transient receptor potential canonical sub-family channel 3 (TRPC3) is considered to play a critical role in calcium homeostasis. However, there are no established findings in this respect with regard to TRPC6. Although the parathyroid gland is a crucial organ in calcium household regulation, little is known about the protein distribution of TRPC channels—especially TRPC3 and TRPC6—in this organ. Our aim was therefore to investigate the protein expression profile of TRPC3 and TRPC6 in healthy and diseased human parathyroid glands. Surgery samples from patients with healthy parathyroid glands and from patients suffering from primary hyperparathyroidism (pHPT) were investigated by immunohistochemistry using knockout-validated antibodies against TRPC3 and TRPC6. A software-based analysis similar to an H-score was performed. For the first time, to our knowledge, TRPC3 and TRPC6 protein expression is described here in the parathyroid glands. It is found in both chief and oxyphilic cells. Furthermore, the TRPC3 staining score in diseased tissue (pHPT) was statistically significantly lower than that in healthy tissue. In conclusion, TRPC3 and TRPC6 proteins are expressed in the human parathyroid gland. Furthermore, there is strong evidence indicating that TRPC3 plays a role in pHPT and subsequently in parathyroid hormone secretion regulation. These findings ultimately require further research in order to not only confirm our results but also to further investigate the relevance of these channels and, in particular, that of TRPC3 in the aforementioned physiological functions and pathophysiological conditions.

## 1. Introduction

The parathyroid gland is an endocrine organ that is allocated fourfold to the posterior surface of the thyroid gland [1]. Its main function is to synthetize and secrete parathyroid hormone, a task which is fulfilled by so-called chief cells [1]. Parathyroid hormone is an important effector in calcium homeostasis, which is basically responsible for increasing serum calcium levels when these are low [1]. Its secretion is dependent upon meticulous serum calcium sensing, ultimately forming a negative feedback loop [2,3]. In addition to the calcium-sensing receptor (CaSR), a homodimeric family C G protein-coupled receptor [4], there is evidence of further molecular players of critical interest in this context. For instance, transient receptor potential canonical subfamily channel 1 (TRPC1) has been demonstrated to be critical for parathyroid hormone release [2,5]. The hypothesis is that increased serum calcium levels activate the CaSR, directly, receptor-operated, or indirectly, store-operated, activating TRPC1 and thus enhancing inhibitory Ca^2+^ influx [2]. Parathyroid chief cells are indeed rather unique in view of the fact that, in most cell types, increased intracellular calcium levels do not inhibit but, on the contrary, actually trigger exocytosis [6]. However, the exact pathway of regulation of parathyroid hormone release remains unknown [3]. In addition to chief cells, so-called oxyphilic cells also exist, although their exact function remains unclarified [7].

The second most frequent endocrinological disease after diabetes mellitus remains primary hyperparathyroidism (pHPT) [8]. Briefly, it is defined by increased serum calcium and parathyroid hormone levels with unknown cause, often found in combination with parathyroid adenomas [9]. Increased calcium levels may have a negative impact on several systems, including the kidneys and bones, and can therefore promote chronic kidney disease [10]. While mild pHPT might not manifest itself clinically for some time, severe pHPT can frequently lead to symptoms such as nausea, dehydration, muscle weakness, cognitive dysfunction, etc. [4].

Although the exact pathophysiology of pHPT is not clear [4,9], there is evidence that TRP channels from the canonical subfamily (TRPC1–7) might be involved [2,5]. They are tetramers, with each monomer being composed of six transmembrane segments and two cytoplasmic domains. The loop between the fifth and sixth transmembrane segments of each monomer contributes to ion pore formation, which is permeable to mono- and bivalent cations [11]. Being finely regulable, TRPC channels, including TRPC3 and TRPC6, are known to act in certain conditions as store- or receptor-operated channels (SOC or ROC) [12]. In the context of calcium homeostasis, TRPC3 has already been shown to play a critical role in renal calcium resorption. For instance, proximal tubular TRPC3 was demonstrated to be nephroprotective in experimental designs of hypercalciuria and nephrolithiasis, as reviewed by Englisch et al. [13]. In this context, we recently presented evidence of the tubular expression of TRPC3 in human specimens [14]. This calcium crosslink between both the kidney and the parathyroid gland [15], and the known potential of TRPC3 to mediate SOC- or ROC-based calcium entry, prompted our interest to investigate TRPC3 protein expression in human parathyroid glands. Indeed, a search in the online library “PubMed” with the keywords “parathyroid” and “TRPC” only provided less than ten results (March 2024), with none of these publications addressing TRPC3 or TRPC6 protein expression in the human parathyroid gland. As a matter of fact TRPC6 possesses abundant protein sequence analogies with TRPC3 [16,17] and displays wide disease implications [13,18]; thus, we aimed to investigate the presence of both TRPC3 and TRPC6 proteins in healthy and diseased human parathyroid tissues. In conclusion, the TRPC3 and TRPC6 proteins are expressed in both chief and oxyphilic cells of the healthy parathyroid gland. Our results further indicate a TRPC3 downregulation in pHPT, ultimately suggesting a critical role for TRPC3 in parathyroid gland physiology and pHPT pathophysiology.

## 2. Materials and Methods

### 2.1. Samples

Parathyroid gland tissue was obtained from surgery specimens, with informed consent provided by human adults who had either been operated due to pHPT (*n* = 4) or due to another cause not affecting the parathyroid gland (*n* = 4). Patient demographics are presented in Table 1. Samples from the pHPT group displayed histologically secured hyperplasia or adenoma and clinically indicated pHPT. The healthy samples displayed no pathologies and were age appropriate as evaluated and labeled by trained pathologists. Three of the samples of healthy parathyroid tissue were obtained from female patients and one from a male patient. The mean age was 48.3 with an SD of 18.9 years at the surgery time point. Samples with pHPT were obtained from four female patients with no known genetic phenotypes of pHPT. The mean age was 52.8 with an SD of 6.3 years at the surgery time point. The research was conducted anonymously, approved by the Ethics Committee of the Saarland Medical Association (130/21), and performed in accordance with the guidelines of the Declaration of Helsinki.

### 2.2. Tissue Treatment

After surgical removal, the parathyroid gland samples were immediately fixed in 4% formalin, where they were kept for 24 h at 4 °C. They were then switched to phosphate-buffered saline for 24 h at 4 °C, exposed to flowing water for 3 h, and incubated in 70% isopropanol for the same period (Otto Fischar GmbH & Co., Saarbrücken, Germany). Afterwards, the following incubation steps were conducted using a mechanical tissue embedder (SLEE medical GmbH, Mainz, Germany): 70% isopropanol (3 h), 80% isopropanol (90 min), 90% isopropanol (90 min), 100% isopropanol (2 × 90 min), methyl benzoate (3 × 90 min; Thermo Fisher Scientific Inc., Waltham, MA, USA), and liquid paraffine (2 × 2 h; Carl Roth GmbH & Co., KG, Karlsruhe, Germany). Later, the samples were sectioned at a thickness of 4 µm and mounted on glass slides.

### 2.3. Histology

Standard hematoxylin and eosin-stained sections were prepared (H&E). Briefly, samples were serially rehydrated through incubation in 100% xylol (15 min; VWR International, Fontenay-sous-Bois, France), followed by decreasing concentrations of isopropanol solutions (100% [10 min], 90% [5 min], 80% [5 min]; Central Chemical Storage, Saarland University, Saarbrucken, Germany), and stained with Ehrlich’s hematoxylin (8 min; Carl Roth GmbH & Co., KG, Karlsruhe, Germany). After washing in distillated water and bluing in fluent water for 12 min, staining with 0.1% eosin (210 s; Central Chemical Storage, Saarland University, Saarbrucken, Germany) was performed and adjusted in 90% isopropanol. Finally, the samples were dehydrated using 100% isopropanol (10 min) and 100% xylol (15 min).

For immunohistochemical staining of TRPC3, TRPC6, and CaSR, the paraffin was removed, and antigen recovery was performed through citrate buffer incubation for 60 min at 95 °C. The samples were incubated with the primary antibody overnight and at room temperature (polyclonal rabbit anti-TRPC3, lyophilized, ACC-016, 1:50, Alomone Labs, Jerusalem BioPark, Jerusalem Israel; polyclonal rabbit anti-TRPC6, lyophilized, ACC-017, 1:50, Alomone Labs, Jerusalem BioPark, Israel; polyclonal rabbit anti-CaSR, lyophilized, ACR-004, 1:100, Alomone Labs, Jerusalem BioPark, Israel). Instead of the primary antibody, 1:500 diluted rabbit serum was used for the negative control. A peroxidase-labeled secondary antibody (HRP, horseradish peroxidase, anti-rabbit goat, A10547; Invitrogen AG, Carlsbad, CA, USA) and diaminobenzidine tetrahydrochloride as chromogen (DAB; incubation time = 3 min for TRPC3 and 6, incubation time = 4 min for CaSR; SK-4103 Vector Laboratories, Burlingame CA, USA) were added to trace the primary antibody. Nuclear counterstaining with hematoxylin followed (C. Roth, Karlsruhe, Germany). The anti-TRPC3, anti-TRPC6, and anti-CaSR antibodies were designed to detect the corresponding channels in mouse, rat, and human tissues, as annotated by the manufacturer (Alomone Labs, Jerusalem BioPark, Israel). Moreover, the anti-TRPC3 antibody (Peptide HKLSEKLNPSVLRC) was designed to detect the amino acid residues 822–835 at the intracellular COOH-terminus of mouse TRPC3, whereas the anti-TRPC6 antibody (Peptide [C]RRNESQDYLLMDELG) detected the amino acid residues 24–38 of the intracelluar N-terminus of mouse TRPC6 (Alomone Labs, Jerusalem BioPark, Jerusalem Israel). The anti-CaSR antibody (Peptide [C]DDYGRPGIEKFREE) recognized the amino acid residues 216–229 at the extracellular N-terminus of human CaSR. The molecular structures of TRPC3 and TRPC6 as well as CaSR are described elsewhere [19,20]. The anti-TRPC antibodies were knockout-validated, as indicated by the manufacturer [21,22,23,24,25].

### 2.4. Evaluation

The Nano Zoomer S210 (Hamamatsu, Japan) was used to digitize the slides. Microphotographs were captured using NDP.view2 image viewing software from Hamamatsu (U12388-01, Hamamatsu, Japan). A semiquantitative analysis of the DAB staining was conducted using Visiopharm image analysis software (version 01.2023, Visiopharm, Denmark). Each slide had a manually annotated region of interest (ROI) with no marginal artefacts. To ensure accurate recognition of histological structures, literature descriptions such as in [1] were consulted and control examinations conducted by trained anatomists.

**Figure 1:**
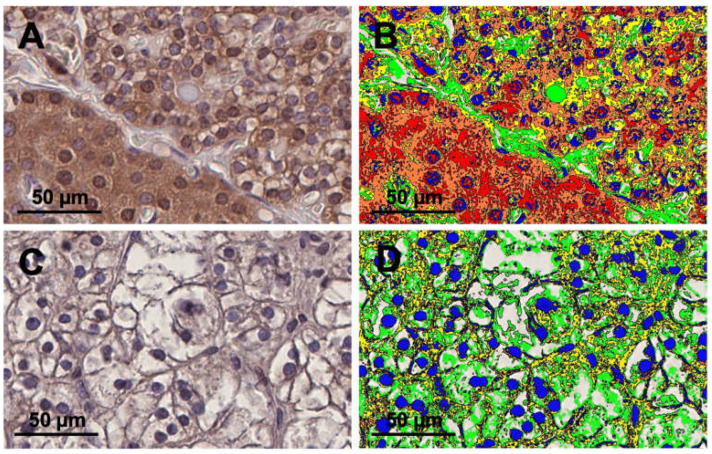
Immunohistochemical staining with an anti-TRPC3 antibody of the human parathyroid gland with and without image analysis. The upper two panels (**A**,**B**) illustrate microphotographs from healthy human parathyroid tissue. The lower two panels (**C**,**D**) present microphotographs from human parathyroid tissue from a patient with primary hyperparathyroidism (pHPT). Panels (**A**,**C**) are without image analysis and panels (**B**,**D**) are with image analysis. Panels (**B**,**D**) show semiquantitative diaminobenzidine tetrahydrochloride (DAB) color scoring. The color red represents a DAB score of 3, orange a DAB score of 2, and yellow a DAB score of 1. The color green represents subtracted connective and fatty tissues. The color blue shows the subtracted hematoxylin area. Both were attributed as DAB-negative. Panels (**A**–**D**) have 40× software magnification and a scale bar of 50 µm.

A threshold system was established to classify the DAB-positive areas within the ROI, whereby the detection of these DAB-positive areas was facilitated by the DAB filter incorporated in Visiopharm software. The threshold value was divided into three sections: between the darkest brown staining (20) and the lightest visible staining (160). Areas with the darkest DAB stain (values between 20–80) were designated as +3, mid-range values (between 81–120) as +2, and the lightest DAB-stained areas (between 121–160) as +1. Furthermore, connective, or fatty tissue was excluded by assigning an extra section (between 0 and 200) with a value of 0–1. These areas, along with hematoxylin staining, were considered DAB-negative. Weighted DAB-positive areas per slide were calculated by multiplying the DAB-positive areas by their respective attributed value (1, 2, or 3) and summing up all values. This value was normalized to the measured tissue area (sum of DAB-negative and DAB-positive areas), resulting in a normalized weighted DAB area score between 0 and 3, akin to a pixelwise H-score (Figure 1) [26]. The positive proportion presented the sum of all DAB-positive areas in relation to the measured tissue area (sum of DAB-positive areas and DAB-negative areas).

### 2.5. Statistical Analysis

Statistical analysis was performed using GraphPad Prism software (version 10.1.0). Descriptive statistics were provided using the mean value and the standard deviation (mean ± SD). The Mann–Whitney *U* test was used for comparisons between two independent cohorts. *p*-values (*p*) are two-sided and were considered significant when <0.05.

## 3. Results

**Figure 2:**
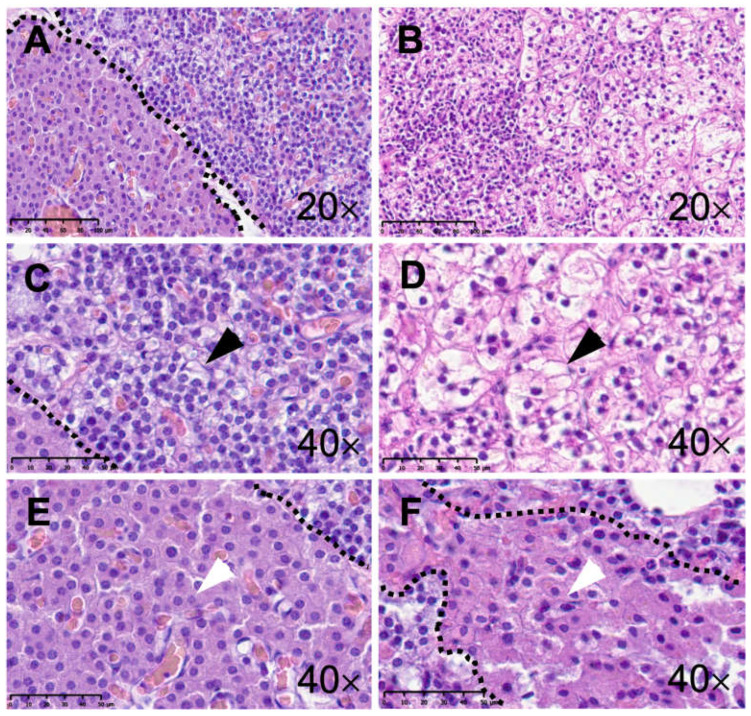
Hematoxylin and eosin staining of human parathyroid tissue. The left column (**A**,**C**,**E**) displays microphotographs from healthy parathyroid tissue and the right column (**B**,**D**,**F**) shows tissue from patients with primary hyperparathyroidism (pHPT). Panel (**A**) features an overview of healthy parathyroid tissue with a pool of chief cells on the right side and a pool of oxyphilic cells on the left side of the demarcation (scale bar: 100 µm). Panels (**C**,**E**) display respectively chief (black arrow) and oxyphilic (white arrow) cells at higher magnification (scale bar: 50 µm). Panel (**B**) displays an overview of human parathyroid tissue from a patient with pHPT (scale bar: 100 µm). Panels (**D**,**F**) display respectively chief (black arrow) and oxyphilic (white arrow) cells at higher magnification (scale bar: 50 µm).

Representative H&E staining of healthy parathyroid tissue as well as tissue from pHPT patients is shown in Figure 2. It displays the morphology of chief and oxyphilic cells in both healthy and pHPT tissues. In the description below, the positive proportion (%) is given first followed by the normalized weighted DAB area score, as detailed in Section 2.4.

**Figure 3:**
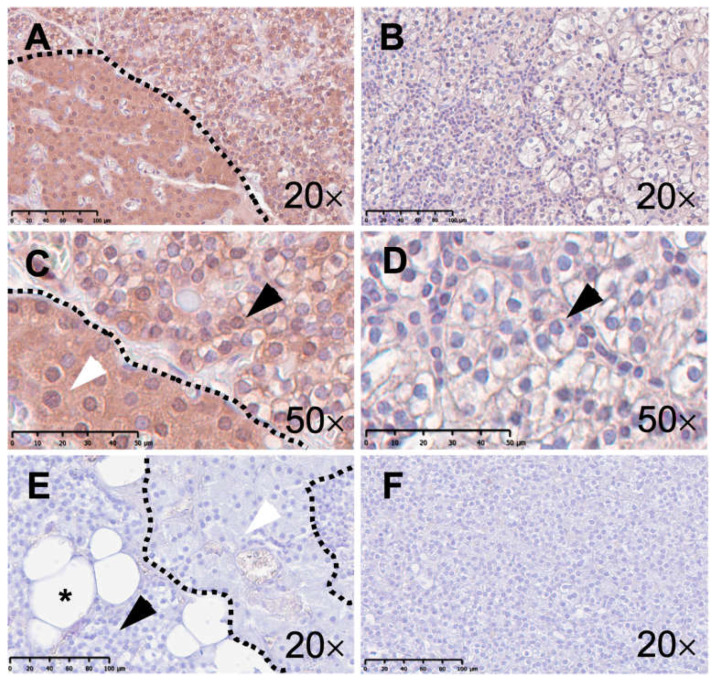
Immunohistochemical staining with an anti-TRPC3 antibody of the human parathyroid gland. The left column (**A**,**C**,**E**) illustrates microphotographs from healthy human parathyroid tissue. The right column (**B**,**D**,**F**) presents microphotographs from human parathyroid tissue from patients with primary hyperparathyroidism (pHPT). Panel (**A**) displays an overview of healthy parathyroid tissue with a pool of chief cells on the right side and a pool of oxyphilic cells on the left side of the demarcation (scale bar: 100 µm). Panel (**C**) displays both chief (black arrow) and oxyphilic (white arrow) cells at higher magnification (scale bar: 50 µm). Panel (**B**) displays an overview of human parathyroid tissue from a patient with pHPT (scale bar: 100 µm). Panel (**D**) displays chief cells (black arrow) at higher magnification (scale bar: 50 µm). Panel (**E**) displays an overview of the negative control staining of healthy parathyroid tissue. Chief (black arrow), oxyphilic (white arrow), and fat cells (asterisk) are displayed (scale bar: 100 µm). Panel (**F**) displays an overview of the negative control staining of parathyroid tissue from a patient with pHPT (scale bar: 100 µm).

Human parathyroid gland tissue displayed immunostaining in all samples after incubation with the anti-TRPC3, anti-TRPC6, and anti-CaSR antibodies. However, obvious contrasts can be described. Healthy parathyroid gland tissue featured anti-TRPC3 staining in both chief and oxyphilic cells in all samples (68 ± 5%; 1.12 ± 0.17; Figure 3A,C). Oxyphilic cells, in particular, revealed pronounced immunoreactivity (Figure 3C). By contrast, all of the samples from pHPT patients presented much weaker immunoreactivity to the anti-TRPC3 antibody (37 ± 7%, *p* = 0.03; 0.44 ± 0.11, *p* = 0.03; Figure 3B,D). Interestingly, one pHPT sample exhibited a somewhat stronger staining pattern than the other three. Only in this sample, oxyphilic cells were detectable, although immunoreactivity to the anti-TRPC3 antibody was still weaker than in healthy parathyroid tissue. Negative controls upon TRPC3 staining displayed no immunoreactivity in both one healthy (4%; 0.04; Figure 3E and one diseased sample (2%; 0.02; Figure 3F).

**Figure 4:**
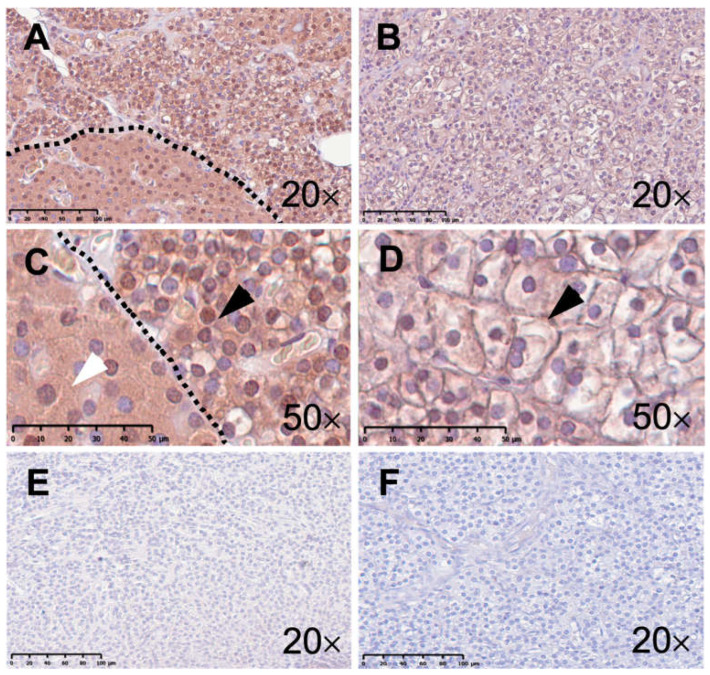
Immunohistochemical staining with an anti-TRPC6 antibody of the human parathyroid gland. The left column (**A**,**C**,**E**) illustrates microphotographs from healthy human parathyroid tissue. The right column (**B**,**D**,**F**) presents microphotographs from human parathyroid tissue from patients with primary hyperparathyroidism (pHPT). Panel (**A**) displays an overview of healthy parathyroid tissue with a pool of chief cells on the right upper side and a pool of oxyphilic cells on the left lower side of the demarcation (scale bar: 100 µm). Panel (**C**) displays both chief (black arrow) and oxyphilic (white arrow) cells at higher magnification (scale bar: 50 µm). Panel (**B**) displays an overview of human parathyroid tissue from a patient with pHPT (scale bar: 100 µm). Panel (**D**) displays chief cells (black arrow) at higher magnification (scale bar: 50 µm). Panel (**E**) displays an overview of negative control staining of healthy parathyroid tissue (scale bar: 100 µm). Panel (**F**) displays an overview of negative control staining of parathyroid tissue from a patient with pHPT (scale bar: 100 µm).

On the other hand, anti-TRPC6 staining was similar in healthy (73 ± 3%; 1.59 ± 0.23; Figure 4A,C) and diseased tissues from pHPT patients (68 ± 6%, *p* = 0.2; 1.36 ± 0.30, *p* = 0.3; Figure 4B,D). Negative controls upon TRPC6 staining displayed no immunoreactivity in both one healthy (3%; 0.03; Figure 4E) and one diseased sample (2%; 0.02; Figure 4F).

**Figure 5:**
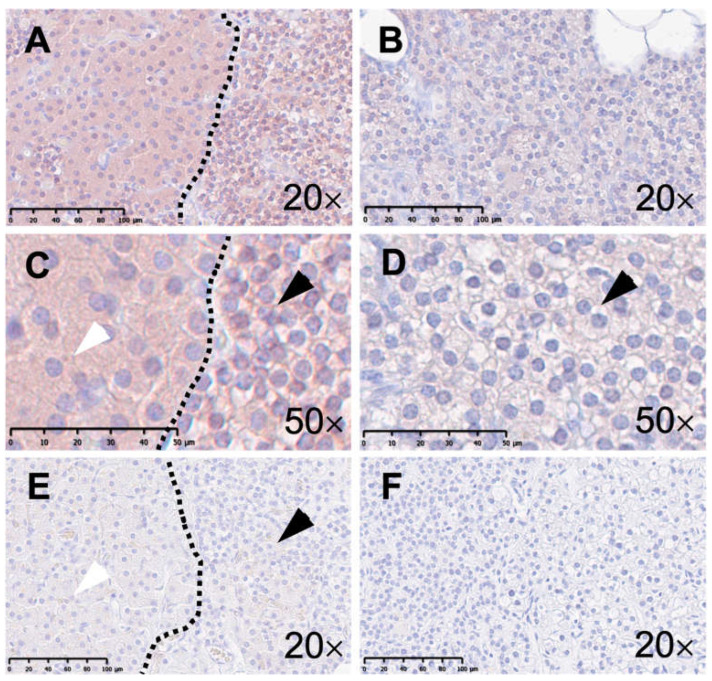
Immunohistochemical staining with an anti-CaSR antibody of the human parathyroid gland. The left column (**A**,**C**,**E**) illustrates microphotographs from healthy human parathyroid tissue. The right column (**B**,**D**,**F**) presents microphotographs from human parathyroid tissue from patients with primary hyperparathyroidism (pHPT). Panel (**A**) displays an overview of healthy parathyroid tissue with a pool of chief cells on the right side and a pool of oxyphilic cells on the left side of the demarcation (scale bar: 100 µm). Panel (**C**) displays both chief (black arrow) and oxyphilic (white arrow) cells at higher magnification (scale bar: 50 µm). Panel (**B**) displays an overview of human parathyroid tissue from a patient with pHPT (scale bar: 100 µm). Panel (**D**) displays chief cells (black arrow) at higher magnification (scale bar: 50 µm). Panel (**E**) displays an overview of the negative control staining of healthy parathyroid tissue. Chief (black arrow) and oxyphilic (white arrow) cells are displayed (scale bar: 100 µm). Panel (**F**) displays an overview of the negative control staining of parathyroid tissue from a patient with pHPT (scale bar: 100 µm).

Additionally, CaSR immunoreactivity was featured in both healthy (Figure 5A,C) and diseased tissues from pHPT patients (Figure 5B,D). Healthy parathyroid tissue showed positive, yet inhomogeneous, immunoreactivity to the anti-CaSR antibody in all samples (45 ± 14%; 0.65 ± 0.32). In contrast, tissue from patients with pHPT demonstrated weaker, yet inhomogeneous, immunoreactivity to the anti-CaSR antibody compared to healthy analogous tissue (21 ± 12%, *p* = 0.03; 0.23 ± 0.13, *p* = 0.03). Negative controls displayed no immunoreactivity in both one healthy (6%; 0.07; Figure 5E) and one diseased sample (3%; 0.03; Figure 5F).

## 4. Discussion

To the best of our knowledge, these findings present—for the first time—evidence indicating wide TRPC3 and TRPC6 protein expression in the human parathyroid gland. This includes both main and oxyphilic cells. We further observed that anti-TRPC3 and anti-CaSR—however not anti-TRPC6—immunolabeling were notably decreased in pHPT-diseased tissue. These findings ultimately suggest that TRPC3 plays a critical role in parathyroid hormone and calcium homeostasis next to the CaSR.

The sample-associated study limitations include the small group size and the respective age and sex variations, factors which possibly restrict the generalizability of our conclusions. Also, protein expression in healthy parathyroid tissue might have been affected by adjacent benign diseased thyroid tissue, although the tissue had been labeled as healthy and age appropriate by trained pathologists.

Immunohistochemistry is a highly specific method that not only enables protein identification but also a detailed histological description of its distribution [27]. However, western blot analysis, which can be restricted in use due to high premises toward the sample’s nature and quality [28,29], could serve to further substantiate the precision of our data. Nevertheless, although immunohistochemistry cannot be quantified and is primarily a qualifying method, tools do exist to perform a semiquantitative analysis [26]. Moreover, such semiquantitative staining analyses are often correlated with western blot data, as observed previously [30,31].

One of the limitations of such a semiquantitative analysis, which could nevertheless affect the evaluation, is variation in sample size. However, there were only minor differences that did not relevantly distort the overall picture. The semiquantitative analysis is intended to be supportive, as, for example, in the case of the H-score, which presents a staining pattern which may correlate with antigen presence but cannot be equated with it [26]. Also, it does not provide any indication of the activity and integrity of the detected protein. 

Interestingly, oxyphilic cells exhibited stronger staining than chief cells, possibly contributing to weaker global staining of diseased tissue given that oxyphilic cells were absent in three of the four pHPT samples. However, chief cells still showed weaker immunolabeling in diseased tissue compared to healthy tissue, making the difference clearly not only attributable to the mismatch in oxyphilic cell presence between the two groups.

In the study presented by Lu et al., reverse transcription polymerase chain reaction (RT-PCR) indicated that *trpc3* complementary deoxyribonucleic acid (cDNA)/messenger ribonucleic acid (mRNA) is not expressed in human parathyroid cells [5]. However, according to the HPA and consensus datasets from the Human Protein Atlas, TRPC3 RNA transcripts can be detected in the parathyroid gland [32,33]. Instead, Lu et al. detected an increase in TRPC1, 4, and 6 mRNA expression in adenomatous compared to healthy parathyroid tissue [5]. A critical limitation of these considerations was a relevant disbalance in cohort size [5]. In this context, it is insightful to observe that, in our study, anti-TRPC6-protein immunolabeling was similar in pHPT tissue in comparison to healthy parathyroid tissue. In contrast, anti-TRPC3 protein immunolabeling was significantly weaker in pHPT tissue compared to healthy tissue. In three of the four pHPT samples, staining seemed almost negative to the human eye, while in the fourth, it was slightly recognizable. Interestingly, anti-TRPC6 immunolabeling was also increased in this sample. This might be attributable to different factors, including artefacts. Following the hypothesis that increased immunostaining is correlated with increased antigen presence, our results would indicate a significant downregulation of TRPC3 protein expression in pHPT. Similar conclusions from immunohistochemistry have been drawn before, but with CaSR [34,35,36]. To our knowledge this has not been described before, all the more in view of the fact that, as previously mentioned, TRPC3 protein expression in the parathyroid gland was unclear. Considering that TRPC3 can act both as SOC and ROC, combined with its relevance in calcium homeostasis, as suggested in the kidney [13,14], a potential role in parathyroid hormone secretion or rather secretion regulation becomes conceivable. The study by Onopiuk et al. did not address TRPC3 but demonstrated that TRPC1 is such a molecular player downstream of CaSR that contributes to the suppression of parathyroid hormone secretion in response to increased serum calcium levels [2]. Inversely, TRPC1 downregulation would be associated with decreased secretion inhibition, as suggested in pHPT. A similar picture emerges from our investigations of human tissue with respect to TRPC3 protein. Moreover, TRPC3 is known to act as a downstream effector of CaSR, as summarized by Englisch et al. [13]. This is further supported by our results that also indicated CaSR downregulation in tissue from pHPT patients. This ultimately suggests an association between the downregulation of TRPC3 and of CaSR, which further underlines the potential relevance of TRPC3, not only in parathyroid physiology but also in pHPT pathophysiology. However, it remains unclear as to whether TRPC3 downregulation is involved in the emergence of pHPT or is only a manifestation of it. It thus becomes even more obvious that, in order to understand the molecular disease dynamics, the impact of serum calcium and parathyroid hormone levels on protein expression, and the function of TRPC3 in the parathyroid gland, further experimental studies are required, which can, for example, include the use of calcimimetics [37].

Overall, further studies with higher case numbers are needed to confirm our results. In addition to the abovementioned experimental investigations, prospective clinical studies with a broad assessment covering a variety of biochemical and clinical data could shed light on the clinical relevance of TRPC3 expression regulation, as already carried out on CaSR [35]. Looking ahead, further differentiating studies are needed on genetic phenotypes and pathological presentations, such as, for example, parathyroid hyperplasia, adenoma, or carcinoma [36].

## 5. Conclusions

The studies by Lu et al. and Onopiuk et al. suggest that there is a clear need for research on the TRPC channels in the parathyroid gland [2,5]. The results we have presented here provide strong evidence for TRPC3 and TRPC6 protein expression in human parathyroid tissue. We describe weaker protein expression of TRPC3—together with CaSR—in tissue from pHPT samples, which suggests a critical function of TRPC3 in pHPT. Further studies are necessary to confirm our findings and to address them both experimentally and in clinical follow-up studies. Ultimately, TRPC3 might become a new target in the prevention or treatment of pHPT.

## Figures and Tables

**Table 1:** Overview of patient demographics. Tissue type (healthy or from primary hyperparathyroidism [pHPT]), age, sex, and surgery indication/pathology are listed for each tissue sample.

Tissue	Age	Sex	Surgery Indication/Pathology
Healthy	23	Female	Hashimoto Thyroiditis (Lymphofollicular Hyperplasia)
Healthy	68	Female	Struma Nodosa (Follicular Lymphocytic Thyroiditis)
Healthy	37	Female	Unspecified Thyroid Pathology
Healthy	65	Male	Struma Multinodosa
pHPT	48	Female	Nodular Hyperplasia of Parathyroid Gland
pHPT	47	Female	Mild diffuse Hyperplasia of Parathyroid Gland
pHPT	63	Female	Parathyroid Adenoma
pHPT	53	Female	Nodular Hyperplasia of Parathyroid Gland

## Data Availability

Data can be obtained from the corresponding author upon reasonable request.

## References

[1] Brown M.B., Limaiem F. (2023). Histology, Parathyroid Gland. StatPearls.

[2] Onopiuk M., Eby B., Nesin V., Ngo P., Lerner M., Gorvin C.M., Stokes V.J., Thakker R.V., Brandi M.L., Chang W. (2020). Control of PTH secretion by the TRPC1 ion channel. JCI Insight.

[3] Brown E.M. (2013). Role of the calcium-sensing receptor in extracellular calcium homeostasis. Best Pract. Res. Clin. Endocrinol. Metab..

[4] Minisola S., Arnold A., Belaya Z., Brandi M.L., Clarke B.L., Hannan F.M., Hofbauer L.C., Insogna K.L., Lacroix A., Liberman U. (2022). Epidemiology, Pathophysiology, and Genetics of Primary Hyperparathyroidism. J. Bone Miner. Res..

[5] Lu M., Branstrom R., Berglund E., Hoog A., Bjorklund P., Westin G., Larsson C., Farnebo L.O., Forsberg L. (2010). Expression and association of TRPC subtypes with Orai1 and STIM1 in human parathyroid. J. Mol. Endocrinol..

[6] Barclay J.W., Morgan A., Burgoyne R.D. (2005). Calcium-dependent regulation of exocytosis. Cell Calcium.

[7] Tanaka Y., Funahashi H., Imai T., Seo H., Tominaga Y., Takagi H. (1996). Oxyphil cell function in secondary parathyroid hyperplasia. Nephron.

[8] Nilsson I.L., Yin L., Lundgren E., Rastad J., Ekbom A. (2002). Clinical presentation of primary hyperparathyroidism in Europe--nationwide cohort analysis on mortality from nonmalignant causes. J. Bone Miner. Res..

[9] Walker M.D., Silverberg S.J. (2018). Primary hyperparathyroidism. Nat. Rev. Endocrinol..

[10] Jha S., Simonds W.F. (2023). Molecular and Clinical Spectrum of Primary Hyperparathyroidism. Endocr. Rev..

[11] Kaneko Y., Szallasi A. (2014). Transient receptor potential (TRP) channels: A clinical perspective. Br. J. Pharmacol..

[12] Abramowitz J., Birnbaumer L. (2009). Physiology and pathophysiology of canonical transient receptor potential channels. FASEB J..

[13] Englisch C.N., Paulsen F., Tschernig T. (2022). TRPC Channels in the Physiology and Pathophysiology of the Renal Tubular System: What Do We Know?. Int. J. Mol. Sci..

[14] Diebolt C.M., Schaudien D., Junker K., Krasteva-Christ G., Tschernig T., Englisch C.N. (2023). New insights in the renal distribution profile of TRPC3—Of mice and men. Ann. Anat..

[15] Moe S.M. (2016). Calcium Homeostasis in Health and in Kidney Disease. Compr. Physiol..

[16] Kiselyov K., Patterson R.L. (2009). The integrative function of TRPC channels. Front. Biosci. (Landmark Ed.).

[17] Wang H., Cheng X., Tian J., Xiao Y., Tian T., Xu F., Hong X., Zhu M.X. (2020). TRPC channels: Structure, function, regulation and recent advances in small molecular probes. Pharmacol. Ther..

[18] Eder P., Groschner K. (2008). TRPC3/6/7: Topical aspects of biophysics and pathophysiology. Channels.

[19] Tang Q., Guo W., Zheng L., Wu J.X., Liu M., Zhou X., Zhang X., Chen L. (2018). Structure of the receptor-activated human TRPC6 and TRPC3 ion channels. Cell Res..

[20] Hendy G.N., Canaff L., Cole D.E. (2013). The CASR gene: Alternative splicing and transcriptional control, and calcium-sensing receptor (CaSR) protein: Structure and ligand binding sites. Best Pract. Res. Clin. Endocrinol. Metab..

[21] Xia W., Wang Q., Lin S., Wang Y., Zhang J., Wang H., Yang X., Hu Y., Liang H., Lu Y. (2023). A high-salt diet promotes hypertrophic scarring through TRPC3-mediated mitochondrial Ca(^2+^) homeostasis dysfunction. Heliyon.

[22] Feng S., Li H., Tai Y., Huang J., Su Y., Abramowitz J., Zhu M.X., Birnbaumer L., Wang Y. (2013). Canonical transient receptor potential 3 channels regulate mitochondrial calcium uptake. Proc. Natl. Acad. Sci. USA.

[23] Harper M.T., Londono J.E., Quick K., Londono J.C., Flockerzi V., Philipp S.E., Birnbaumer L., Freichel M., Poole A.W. (2013). Transient receptor potential channels function as a coincidence signal detector mediating phosphatidylserine exposure. Sci. Signal.

[24] Kistler A.D., Singh G., Altintas M.M., Yu H., Fernandez I.C., Gu C., Wilson C., Srivastava S.K., Dietrich A., Walz K. (2013). Transient receptor potential channel 6 (TRPC6) protects podocytes during complement-mediated glomerular disease. J. Biol. Chem..

[25] Nakhoul N.L., Tu C.L., Brown K.L., Islam M.T., Hodges A.G., Abdulnour-Nakhoul S.M. (2020). Calcium-sensing receptor deletion in the mouse esophagus alters barrier function. Am. J. Physiol. Gastrointest. Liver Physiol..

[26] Ram S., Vizcarra P., Whalen P., Deng S., Painter C.L., Jackson-Fisher A., Pirie-Shepherd S., Xia X., Powell E.L. (2021). Pixelwise H-score: A novel digital image analysis-based metric to quantify membrane biomarker expression from immunohistochemistry images. PLoS ONE.

[27] Magaki S., Hojat S.A., Wei B., So A., Yong W.H. (2019). An Introduction to the Performance of Immunohistochemistry. Methods Mol. Biol..

[28] Pillai-Kastoori L., Schutz-Geschwender A.R., Harford J.A. (2020). A systematic approach to quantitative Western blot analysis. Anal. Biochem..

[29] Taylor S.C., Posch A. (2014). The design of a quantitative western blot experiment. Biomed. Res. Int..

[30] Lang H.B., Xie R.X., Huang M.L., Fang L.Y., Tang Y.B., Zhang F. (2020). The Effect and Mechanism of TRPC1, 3, and 6 on the Proliferation, Migration, and Lumen Formation of Retinal Vascular Endothelial Cells Induced by High Glucose. Ophthalmic Res..

[31] Du Y., Fu J., Yao L., Qiao L., Liu N., Xing Y., Xue X. (2017). Altered expression of PPAR-gamma and TRPC in neonatal rats with persistent pulmonary hypertension. Mol. Med. Rep..

[32] Uhlen M., Fagerberg L., Hallstrom B.M., Lindskog C., Oksvold P., Mardinoglu A., Sivertsson A., Kampf C., Sjostedt E., Asplund A. (2015). Proteomics. Tissue-based map of the human proteome. Science.

[33] https://www.proteinatlas.org/ENSG00000138741-TRPC3/tissue/parathyroid+gland.

[34] Agarwal S., Kardam S., Chatterjee P., Kumar C., Boruah M., Sharma M.C., Tabin M., Ramakrishnan L. (2022). CaSR expression in normal parathyroid and PHPT: New insights into pathogenesis from an autopsy-based study. J. Endocrinol. Investig..

[35] Sengul Aycicek G., Aydogan B.I., Sahin M., Cansiz Ersoz C., Sak S.D., Baskal N. (2018). Clinical Impact of p27(Kip1) and CaSR Expression on Primary Hyperparathyroidism. Endocr. Pathol..

[36] Worth A.L., Ayrapetyan M., Maygarden S.J., Li Z., Wu Z., Agala C.B., Kim L.T. (2024). Expression of the Calcium-Sensing Receptor on Normal and Abnormal Parathyroid and Thyroid Tissue. J. Surg. Res..

[37] Kawata T., Imanishi Y., Kobayashi K., Kenko T., Wada M., Ishimura E., Miki T., Nagano N., Inaba M., Arnold A. (2005). Relationship between parathyroid calcium-sensing receptor expression and potency of the calcimimetic, cinacalcet, in suppressing parathyroid hormone secretion in an in vivo murine model of primary hyperparathyroidism. Eur. J. Endocrinol..

